# Long-term Exposure to Black Carbon and Carotid Intima-Media Thickness: The Normative Aging Study

**DOI:** 10.1289/ehp.1104845

**Published:** 2013-07-02

**Authors:** Elissa H. Wilker, Murray A. Mittleman, Brent A. Coull, Alexandros Gryparis, Michiel L. Bots, Joel Schwartz, David Sparrow

**Affiliations:** 1 Cardiovascular Epidemiology Research Unit, Beth Israel Deaconess Medical Center, Boston, Massachusetts, USA; 2 Department of Environmental Health; 3 Department of Epidemiology, and; 4 Department of Biostatistics, Harvard School of Public Health, Boston, Massachusetts, USA; 5 Julius Center for Health Sciences and Primary Care, Utrecht, the Netherlands; 6 Normative Aging Study, Veterans Affairs Boston Healthcare System, Boston, Massachusetts, USA; 7 Department of Medicine, Boston University School of Medicine, Boston, Massachusetts, USA

## Abstract

Background: Evidence suggests that air pollution is associated with atherosclerosis and that traffic-related particles are a particularly important contributor to the association.

Objectives: We investigated the association between long-term exposure to black carbon, a correlate of traffic particles, and intima-media thickness of the common carotid artery (CIMT) in elderly men residing in the greater Boston, Massachusetts, area.

Methods: We estimated 1-year average exposures to black carbon at the home addresses of Normative Aging Study participants before their first CIMT measurement. The association between estimated black carbon levels and CIMT was estimated using mixed effects models to account for repeated outcome measures. In secondary analyses, we examined whether living close to a major road or average daily traffic within 100 m of residence was associated with CIMT.

Results: There were 380 participants (97% self-reported white race) with an initial visit between 2004 and 2008. Two or three follow-up CIMT measurements 1.5 years apart were available for 340 (89%) and 260 (68%) men, respectively. At first examination, the average ± SD age was 76 ± 6.4 years and the mean ± SD CIMT was 0.99 ± 0.18 mm. A one-interquartile range increase in 1-year average black carbon (0.26 µg/m^3^) was associated with a 1.1% higher CIMT (95% CI: 0.4, 1.7%) based on a fully adjusted model.

Conclusions: Annual mean black carbon concentration based on spatially resolved exposure estimates was associated with CIMT in a population of elderly men. These findings support an association between long-term air pollution exposure and atherosclerosis.

Citation: Wilker EH, Mittleman MA, Coull BA, Gryparis A, Bots ML, Schwartz J, Sparrow D. 2013. Long-term exposure to black carbon and carotid intima-media thickness: the Normative Aging Study. Environ Health Perspect 121:1061–1067; http://dx.doi.org/10.1289/ehp.1104845 [Online 2 July 2013]

## Introduction

Exposure to particulate air pollution has been associated with cardiovascular morbidity and mortality in numerous epidemiologic studies (Brook 2004; Brook et al. 2010; Dockery et al. 1993; Pope and Dockery 2006). Evidence suggests that local traffic is a major source of within-city heterogeneity in air pollution exposures (Brugge et al. 2007; Clougherty et al. 2008) and that mobile sources of pollution may be an important contributor to adverse health effects (Künzli et al. 2000; Laden 2000; Peters et al. 2004). Several studies focusing specifically on the traffic-related constituents of pollution have reported short-term associations with indicators of cardiovascular health (Delfino et al. 2010a, 2010b; Madrigano et al. 2010; Mordukhovich et al. 2009). Evidence for long-term effects of chronic exposure to traffic-related air pollution has come largely from animal studies, which have demonstrated proatherosclerotic effects of diesel exhaust particles and concentrated ambient urban particles (Chen and Nadziejko 2005; Quan et al. 2010; Sun 2005). Recently, a growing number of epidemiologic studies have also observed associations between subclinical atherosclerosis and estimated fine particulate matter (particulate matter ≤ 2.5 µm in aerodynamic diameter; PM_2.5_) or distance to major roadway (Bauer et al. 2010; Diez Roux et al. 2008; Hoffmann et al. 2007; Künzli et al. 2005).

Black carbon is a correlate of traffic-related combustion products, and a common surrogate for traffic particles in general, weighted toward diesel particles. We have developed a nonlinear land use regression model to estimate black carbon exposures and have applied it within the greater Boston, Massachusetts, metropolitan area (Gryparis et al. 2007). In the present study making use of up to three repeated carotid intima-media thickness (CIMT) measures in a cohort of elderly men, we hypothesized that the estimated annual average concentration of black carbon at participants’ homes in the year before the first study visit would be associated with CIMT, a reliable measure of subclinical atherosclerosis (Kanters et al. 1997; O’Leary and Bots 2010) that predicts cardiovascular outcomes (Nambi et al. 2012; O’Leary et al. 1999). In secondary descriptive analyses, we also estimated associations with residential proximity to a major roadway [defined as U.S. Census feature Class Code A1 (Primary Highway with Limited Access) or A2 (Primary Highway Without Limited Access)] and with average daily traffic within 100 m of residence.

## Methods

**Figure 1:**
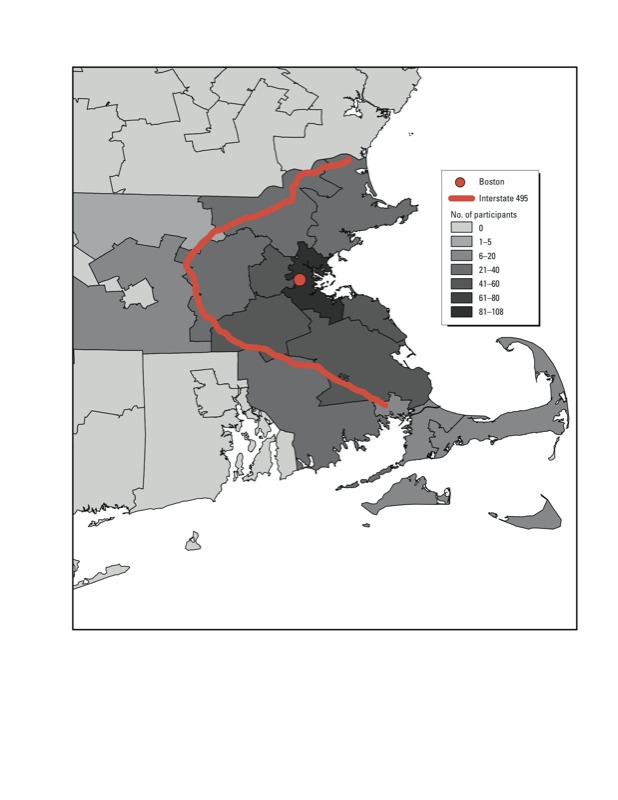
Number of participants according to baseline residence locations within 3-digit ZIP codes in Massachusetts. Most participants lived in the greater Boston area.

*Study population.* The Normative Aging Study is a cohort of community-dwelling men from the greater Boston area recruited in the early 1960s. CIMT was measured in a subsample of participants beginning in 2004, after participants had been followed for four decades. Participants in the CIMT substudy were followed for up to three time points scheduled 1.5 years apart. Our analysis included 380 participants with complete information regarding black carbon concentrations and all covariates at baseline (i.e., the time of the first CIMT measurement). Baseline visits occurred between 2004 and 2008, and there were a total of 980 examinations between 2004 and 2010. All participants gave written informed consent prior to initiation of the study, and the study was approved by the institutional review boards of all participating institutions. A map of the region of participants’ locations of residences is provided in Figure 1.

*Physical parameters and medical history.* Study center visits were subsquent to an overnight fast and abstention from smoking. Physical examinations included height and weight measurements, and body mass index (BMI) was calculated as weight in kilograms divided by height in meters squared. We used questionnaires to evaluate smoking habits and medication use, with responses confirmed by an on-site physician. Alcohol intake (servings per day) was determined using the Cornell Medical Index (Brodman et al. 1949). Overall physical activity was assessed as metabolic equivalent of task (METs) per week.

*CIMT measurements.* We assessed CIMT using a high-resolution ultrasound scanner (Toshiba Powervision 6000 SSA-370A; Toshiba America Medical Systems, Tustin, CA, USA) equipped with a 7.5-MHz linear array transducer. Images were frozen on the top of the R-wave of the electrocardiogram and recorded on super VHS tape. The widths of the near and far walls of the distal 10 mm of the right and left common carotid arteries were measured from three predefined angles of measurement (90°, 120°, and 150° on the right side; 210°, 240°, and 270° on the left side) using an automated edge detection program called the Artery Measurement System (Liang et al. 2000). The three measurements were averaged for each segment, and the four resulting values (i.e., left far, left near, right far, and right near segments, respectively) were averaged to calculate the overall mean CIMT for each participant at each visit. Estimates of left far, left near, right far, and right near CIMT were available on 97% of the 380 participants at the initial baseline assessment. If a segment measurement was missing, the mean of available estimates was used. All scans were performed at the Veterans Affairs Boston Healthcare System using the same ultrasound machine and identical protocols for scanning and reading of images. Reproducibility of these methods for these approaches has been published previously (Dogan et al. 2010).

*Individual-level black carbon estimates and meteorological measurements.* We have built, validated, and published a spatiotemporal model for 24-hr estimates of black carbon based on > 6,021 observations from > 2,079 unique exposure days at 82 locations in the greater Boston area (Gryparis et al. 2007). Predictions are based on meteorological and other characteristics (e.g., weekday vs. weekend) of a particular day, as well as measures of land use (e.g., cumulative traffic density within 100 m, population density, distance to nearest major roadway, percent urbanization) at a given location. Weather data were obtained from the Boston airport weather station, and planetary boundary layer data from the National Oceanic and Atmospheric Administration’s national reanalysis data set (http://www.esrl.noaa.gov/psd/data/gridded/data.narr.html).

Measurements from continuous ambient monitors and specific monitoring campaigns were used as to develop model predictions. We obtained hourly outdoor black carbon concentrations from a total of 15 ambient monitors from 1999 to 2004: 12 of these monitors were included in a study of spatial variability in traffic-related pollutant concentrations conducted by the Northeast States for Coordinated Air Use Management (NESCAUM), two sites were operated by the Massachusetts Department of Environmental Protection, and one site was located on the roof of Harvard School of Public Health (HSPH) and operated by the HSPH Department of Environmental Health. Measurements from specific monitoring campaigns came from two sources. First, beginning in 1999, hourly outdoor and indoor black carbon concentrations were measured inside and outside of 30 residential homes, using aethelometers over 48-hr intervals as part of a National Institute of Environmental Health Sciences (NIEHS)–funded study of air pollution and heart rate variability (APAHRV) conducted at HSPH (Zanobetti et al. 2010). Second, 24-hr average outdoor elemental carbon concentrations measured over 7-day periods in 23 locations during the winter and summer of 2000 were obtained from a U.S. Environmental Protection Agency (EPA)–funded multipollutant exposures study of sensitive individuals (Brown et al. 2008). We have also incorporated interactions between temporal predictors, such as mixing height and wind speed, and source-based geographic variables, such as traffic density. We used regression splines to allow nonlinear relations between predictors and exposure levels and thin-plate splines (a two-dimensional extension of regression splines) to model spatial variability that was not accounted for by the spatial predictors in the model. Separate models were fit for warm and cold seasons. The prediction equation also used central monitor concentrations as an independent variable to reflect average concentration levels for a given day. In essence, the central monitor concentrations serve as direct estimates of the daily time effect. Latent variable models were used to integrate measures of outdoor black carbon, indoor black carbon, and elemental carbon from the monitoring campaigns. The resulting multipollutant model is fit using a Bayesian Markov Chain Monte Carlo approach. The adjusted *R*^2^ for the model was 0.83 and the average correlation between predicted and measured BC concentrations in four out-of-sample validation samples was 0.59. This model has been previously used to examine the associations between black carbon and a variety of health outcomes including mortality (Maynard et al. 2007), cognitive function in children (Suglia et al. 2008) and adults (Power et al. 2011), birth weight (Gryparis et al. 2009), lung function (Franco Suglia et al. 2008), and biomarkers of endothelial function (Alexeeff et al. 2011).

GIS (geographic information system) analyses were performed using ArcMap, version 10.1 (ESRI, Redlands CA, USA). All addresses of Normative Aging Study participants have been geocoded and assigned daily predicted black carbon concentrations. For this analysis, we calculated annual average concentrations during the year before the first CIMT measurement for participants for ≥ 9 months of contiguous daily black carbon data during that year. Available data were used as a proxy for a 1-year average for participants who were missing 1–3 months of data. The primary reasons that individuals did not have complete black carbon data was moving to a new home or having a second home outside the study region. If an individual had multiple addresses during the year before their first CIMT measurement and black carbon data were available for all addresses, a composite individual-level annual mean measure of black carbon was calculated using data from the time the individual spent at each address.

*Distance to roadway measures and average daily traffic within 100 m.* We also examined associations with distance to a major roadway and traffic density within a 100-m buffer of residence during the year before the first CIMT measurement in descriptive analyses. Major roadway classifications were provided by Census TIGER files (U.S. Census Bureau 2013). Distance to the nearest major roadway (Class Code A1 or A2) was computed using the near tool in ArcGIS. We classified residential distance to major roadways as 0 to ≤ 100 m, 100 to ≤ 200 m, 200 to ≤ 1,000 m, and > 1,000 m based on prior studies showing that living within 100 m of a major road is associated with cardiovascular events (Tonne et al. 2007), all-cause mortality among myocardial infarction survivors (Rosenbloom et al. 2012), and atherosclerotic progression (Künzli et al. 2010). In addition, living ≤ 200 m of a major road has been associated with coronary artery calcification, another measure of atherosclerotic burden (Hoffmann et al. 2007). Average daily traffic was determined by summing the product of road segment length and estimated annual average daily traffic within a 100-m buffer of the subject’s residence using the MassHighway 2002 Road Inventory database (Massachusetts Department of Transportation 2013). The measures of annual average daily traffic are based on actual traffic counts for major roadways and estimated according to regional traffic for more local roads.

*Statistical methods.* CIMT was natural log (ln) transformed to improve the normality of the dependent variable distribution. We fit general additive models based on penalized splines using both ln-transformed and untransformed annual average black carbon concentrations to assess the functional form of the outcome–exposure association. These models strongly supported a linear association between CIMT and ln-transformed black carbon exposure, whereas the spline on the natural scale is reported in the Supplemental Material, Figure S1, and showed a nonlinear association. Specifically, there was a positive association between black carbon and CIMT at lower levels of black carbon, where most of the observations occurred, and a negative association with wide confidence intervals (CIs) at high levels, where there were few observations. Therefore, our primary analyses were based on ln-transformed black carbon exposures. We estimated the association between ln-transformed CIMT and annual average ln-transformed daily black carbon concentrations at each participant’s home address during the year before their first CIMT measurement using linear mixed effects with subject-specific random intercepts in SAS PROC MIXED (SAS, version 9.1; SAS Institute Inc., Cary, NC, USA) to account for repeated measures of the outcome in a subset of participants.

We fit our models in stages, first adjusting for a limited set of covariates including age at baseline, BMI, smoking status, and time since beginning of the study to capture long-term time trends. In the second stage, we added information on health status, medication use, and socioeconomic position, any of which could be potential confounders or mediators of the association between black carbon exposure and atherosclerosis. Covariates included in the second stage model were pack-years smoked, years of education (< 12, 12–16, and > 16 years), alcohol intake (≥ 2 servings/day), categories of physical activity (0 to < 12, 12 to < 30, ≥ 30 METs/week), median income of the 2000 U.S. Census tract of residence, total cholesterol, lipid-lowering medication (statins), systolic blood pressure, blood pressure medications, and diabetes diagnosis. Time-varying covariates (all but education, baseline exposure, and median income at the U.S. Census tract at baseline) were updated at each visit. We also examined effect modification by using cross-product terms to estimate associations between black carbon and CIMT stratified on age (≤ 75, > 75 years of age), education (≤ 12, > 12 years of education), statin use (yes or no), diabetes (yes or no), and obesity (BMI < 30, ≥ 30) at baseline.

We conducted a separate analysis of CIMT progression (specifically, the change in CIMT from baseline to 3 years after baseline) in association with ln-transformed black carbon at baseline among the 260 participants with a measure of CIMT after 3 years of follow up, adjusting for the baseline values of covariates included in the fully adjusted model.

We also conducted several sensitivity analyses. We examined whether removal of potential confounders and mediators (i.e., statin medication use, total cholesterol, blood pressure medication use, diabetes diagnosis) altered the findings, and we also removed age at baseline from our model because some studies have observed that age is an important confounder and potential effect modifier (Rivera et al. 2013). Next, we explored the exposure–response relationship in fully adjusted models using generalized additive models with penalized splines in R (version 2.13; R Foundation for Statistical Computing, Vienna, Austria). We also estimated the cross-sectional association between black carbon during the year before the first CIMT measurement, and CIMT at the first measurement, with model covariates as defined at baseline. We then restricted analyses to participants whose location of residence did not change during the study and examined associations with a 1-year average exposure to black carbon in 2003 as a surrogate for long-term exposure among this population. The year 2003 was chosen because it is the year before the earliest possible baseline visits. In addition, we estimated the association between black carbon and CIMT after excluding participants who resided outside of the eastern Massachusetts region [approximately within the boundaries of Interstate 495 (I-495); Figure 1] where the original exposure model was developed and tested. We also considered clustering by socioeconomic status by including an additional random effect for ZIP code. In secondary descriptive analyses, we estimated associations between CIMT and residential proximity to a major roadway (0 to ≤ 100 m, > 100 to ≤ 200 m, > 200 to ≤ 1,000 m, or > 1,000 m) and between CIMT and average daily traffic within 100 m of the participant’s residence (categorized according to quartiles).

## Results

A total of 380 participants completed a baseline visit between 2004 and 2008, including 340 (89%) who completed at least two follow-up visits and 260 (68%) who completed three follow-up visits through 2010 (Table 1). At baseline (the time of first CIMT measure), the mean (± SD) age and BMI of participants were 75.9 ± 6.4 years and 28.1 ± 4.1 kg/m^2^ respectively, and 355 (93%) were retired or semiretired. The mean CIMT at baseline was 0.99 ± 0.18 mm, which was higher than levels observed in younger, population-based cohorts. Participants were 61 to 96 years of age at baseline and CIMT ranged from 0.63 to 2.10. Median predicted black carbon concentrations at baseline for each subject was 0.29 µg/m^3^, with the interquartile range (IQR) equivalent to 0.26 µg/m^3^ (25th to 75th quartile, 0.16–0.42 µg/m^3^).

**Table 1:** Population characteristics [mean ± SD or *n* (%)] at study center visits (2004–2008) when CIMT was measured.

Characteristic	Visit 1 (*n* = 380)	Visit 2 (*n* = 340)	Visit 3 (*n* = 260)
Age (years)	75.9 ± 6.4	77.1 ± 6.2	78.0 ± 6.0
BMI (kg/m^2^)	28.1 ± 4.1	28.2 ± 4.2	28.1 ± 4.2
Median income (US$)	63,334 ± 20,196	63,360 ± 20,353	64,286 ± 20,858
Pack-years	17.8 ± 22.7	18.2 ± 23.5	15.9 ± 19.9
METs (hr/week)			
< 12	247 (65)	215 (63)	163 (63)
12–< 30	81 (21)	74 (22)	52 (20)
≥ 30	50 (13)	50 (15)	45 (17)
Education (years)			
< 12	6 (2)	5 (2)	2 (1)
12–16	215 (57)	190 (56)	148 (57)
> 16	159 (42)	145 (43)	110 (42)
Cholesterol (mg/dL)	174 (37)	173 (37)	170 (34)
Statin use	208 (55)	202 (59)	162 (62)
Type 2 diabetes	74 (20)	72 (21)	59 (23)
≥ 2 Servings of alcohol per day	67 (18)	60 (18)	46 (18)
CMIT (mm)	0.99 ± 0.18	1.00 ± 0.18	1.01 ± 0.18
Years since baseline	0	1.6 ± 0.4	3.0 ± 0.2
Progression since baseline (mm)	0	0.02 ± 0.08	0.04 ± 0.09

Based on our fully adjusted model, an IQR increase in annual average black carbon during the year before baseline was associated with a 1.1% (95% CI: 0.4, 1.7%) higher CIMT, consistent with a 0.01-mm increase from the mean CIMT of 0.99 mm at baseline (Table 2), which was similar to the mean rate of progression per year in this population (i.e., 0.01 mm). This association was stronger than estimated by the parsimonious model (0.9% higher; 95% CI: 0.2, 1.5%) adjusted for age, BMI, smoking, and time since baseline only.

**Table 2:** CIMT percent difference associated with a 1-IQR increase (0.26 µg/m^3^) in average exposure to black carbon during the year before the first CIMT measurement.

Modeling approach	Percent difference (95% CI)	No. of participants	No. of observations
Model
Parsimonious model (model 1)^*a*^	0.9 (0.2, 1.5)	380	977
Fully adjusted model (model 2)^*b*^	1.1 (0.4, 1.7)	378	968
Sensitivity analyses
Age + other potential mediators removed^*c*^	1.2 (0.5, 1.9)	380	976
Medications and health covariates only^*d*^	0.8 (0.2, 1.5)	380	971
Cross-sectional model^*e*^	1.0 (0.3, 1.7)	378	378
Stable residential address only^*f*^	1.0 (0.3, 1.7)	320	824
Black carbon in 2003—stable residential address for entire study^*f*^	0.8 (0.2, 1.5)	315	816
Restriction to region where model was originally built and validated for whole population^*f*^	1.4 (–1.3, 4.2)	295	750
Restriction to region where model was originally built and validated stable residential address for entire study^*f*^	1.9 (–1.0, 4.9)	254	646
Random effect for ZIP code^*f*^	1.1 (0.4, 1.8)	378	968
^***a***^Adjusted for age at baseline, BMI, smoking status (never, current, former), time since baseline (days). ^***b***^Adjusted for model 1 covariates + statin medication use, total cholesterol, systolic blood pressure, blood pressure medication use, and diabetes diagnosis, pack-years smoked, level of education, ≥ 2 servings of alcohol per day, physical activity (METs per week: 0 to < 12,12 to < 30, ≥ 30 hr), and U.S. Census tract–level median income. ^***c***^Adjusted for BMI, pack-years smoked, smoking status, education status, ≥ 2 servings of alcohol per day, median income, time since study baseline. ^***d***^Adjusted for age at baseline, BMI, pack-years smoked, statin use, blood pressure medications, smoking status, diabetes diagnosis, adjusted exam date, systolic blood pressure, and total cholesterol. ^***e***^Adjusted for age at baseline, BMI, pack-years smoked, statin use (yes/no), blood pressure meds (yes/no), smoking status (never, current, former), education status (< 12 years, 12–16, > 16 years), ≥ 2 servings of alcohol per day, median income at the census tract level, diabetes (yes/no), time since study inception, systolic blood pressure, cholesterol. ^***f***^Adjusted for model 2 covariates.			

*p*-Values for cross-product terms indicated a statistically significant interaction (*p* < 0.05) for BMI only (Table 3). Specifically, the association between an IQR increase in black carbon and CIMT was stronger among men who were not obese at baseline (1.4%; 95% CI: 0.7, 2.2%; *n* = 284) than among men who were obese (0.6%; 95% CI: –0.6, 1.8%; *n* = 96, *p*_interaction_ = 0.04). No other significant interactions were observed. CIMT progression among the 260 men with CIMT measured 3 years apart was not significantly associated with an IQR increase in annual average black carbon at baseline (–0.0002 mm decrease from baseline on average; 95% CI: –0.002, 0.001 mm; *p* = 0.79)

**Table 3:** Model 2 results stratified by potential susceptibility factors according to potential effect modifiers at baseline.

Stratification factor	Category	Percent difference (95% CI)	*p*_interaction_
Age	≤ 75 years	0.9 (–0.04, 1.7)	0.33
	> 75 years	1.5 (0.6, 2.5)
Education	≤ 12 years	1.1 (0.1, 2.0)	0.75
	> 12 years	1.1 (0.2, 2.0)
Statin use	No	1.2 (0.1, 2.2)	0.97
	Yes	0.9 (0.2, 1.7)
BMI > 30	No	1.4 (0.7, 2.7)	0.04
	Yes	0.6 (–0.6, 1.8)
Diabetes	No	1.1 (–0.4, 1.7)	0.28
	Yes	0.9 (–1.1, 2.8)
At first visit, there were 213 individuals > 75 years of age (*p* = 0.33), 96 obese individuals (*p* = 0.04), 241 participants with education beyond high school (*p* = 0.75), 208 using statins (*p* = 0.97), and 74 with diabetes (*p* = 0.28).			

In our sensitivity analyses (Table 2), point estimates did not change when we removed additional potential mediators associated with health status. Results from our cross-sectional analysis restricted to first measure of CIMT produced similar estimates to those observed in our final model. We did not observe any deviation from linearity on the log scale. In analyses in which we excluded participants who changed their residence during the study (*n* = 60, 16%), results were similar to final models. Examining the association among individuals with stable residence since 2003 produced similar, slightly attenuated estimates. A 2.4-fold difference in exposure in 2003 (median = 0.30 µg/m^3^, IQR = 0.19–0.46) was associated with 0.8% higher CIMT (95% CI: 0.1, 1.5%). In analyses in which we additionally excluded individuals with addresses outside the region in which the model was originally developed and tested, point estimates were stronger but did not meet statistical significance because of the reduction in sample size. Inclusion of the random effect for ZIP code did not materially change the results from the full model [1.1% higher CIMT (95% CI: 0.4, 1.8%)].

In descriptive analyses, we estimated associations between residential proximity to an A1 or A2 roadway and average daily traffic within 100 m of home address at baseline. Because there were only 10 participants living < 100 m from a major road, we collapsed the ≤ 100-m and 100- to ≤ 200-m categories. In comparison to living > 1,000 m from a major road, living < 200 m or 200–1,000 m away was associated with –1.4% (95% CI: –7.11, 4.6%) or –2.2% lower CIMT (95% CI: –5.7, 1.5%), respectively. For the average daily traffic, we estimated associations by quartiles of exposure (7,795–212,923; 212,924–416,029; 416,030–1,251,886; and 1,251,887–9,861,107 vehicles/day). Compared with living in a location in the lowest quartile of exposure, living in a location with average daily traffic in the second, third, or fourth quartile of exposure was associated with 4.6% higher (95% CI: –0.3, 9.6%), 4.3% higher (95% CI: –0.5, 9.3%), or 2.7% (95% CI: –2.0, 7.6%) higher CIMT, respectively.

## Discussion

We observed a positive association between average black carbon exposure at the home address during the year before baseline and subsequent subclinical atherosclerosis measured by CIMT. A major feature of this study is the use of modeled black carbon at home address; black carbon is a more specific marker for traffic particles than PM_2.5_ and one which can show considerable spatial heterogeneity within distances of a few hundred meters. Our findings are consistent with prior studies from cross-sectional analyses of CIMT and PM_2.5_ exposures among participants in Los Angeles, California (Künzli et al. 2005); the Multi-Ethnic Study of Atherosclerosis (MESA) (Diez Roux et al. 2008), which used 20-year estimates of modeled exposure; and 1-year averaged modeled exposure from the Heinz-Nixdorf Recall study in Germany (Bauer et al. 2010) in addition to a recent study that reported associations with NO_2_ (nitrogen dioxide), which is also a marker of traffic particles (Rivera et al. 2013).

Previous studies have reported that CIMT is a strong predictor of future vascular events. A meta-analysis reported that age- and sex- adjusted relative risks of myocardial infarction and stroke per 0.10 mm CIMT difference were 1.15% (95% CI: 1.12, 1.17%) and 1.18% (95% CI: 1.16, 1.21%), respectively; and although the relationship between CIMT and risk was not linear, linear models fit well for moderate-to-high CIMT levels. The levels of CIMT at baseline observed for men who participated in our study were higher than those reported in populations that included younger, healthier populations of both men and women (Chambless et al. 1997; Lim et al. 2008) and were more similar to those reported in a study of patients with arterial disease and cardiovascular risk factors (Dijk et al. 2006). In the Atherosclerosis Risk in Communities (ARIC) study, a cutoff of 1 mm was used to evaluate risk of coronary heart disease, and the hazard ratio for coronary heart disease comparing men with CIMT measures ≥ 1 mm versus < 1 mm was 1.85 (95% CI: 1.28, 2.69), which would suggest that the levels of CIMT observed in our study population might be associated with elevated risk of cardiovascular disease.

A few recent studies have begun to examine associations between ambient air pollution exposure and atherosclerotic progression as measured by CIMT. In one recent study, both PM_2.5_ and residential distance from highway were examined in a population of participants pooled from five double-blind randomized trials that estimated the effects of interventions on the progression of CIMT in the Los Angeles area (Künzli et al. 2010). Künzli et al. (2010) observed a rate of progression of 5.46 µm (95% CI: 0.13, 10.79) per year associated with living within 100 m of major road and that a 10-µg/m^3^ increase in PM_2.5_ was associated with a slightly smaller change of 2.53 (95% CI: –0.31, 5.38) µm per year. More recently, similar results have been reported in MESA, where Adar et al. (2013) observed that 2.5-µg/m^3^ higher levels of residential PM_2.5_ during the follow-up period were associated with 5.0 µm/year (95% CI: 2.6, 7.4 µm/year) greater IMT progressions among persons in the same metropolitan area. We did not observe a significant association with progression over 3 years; given the relatively short period of follow-up on only 260 participants, we had limited power to test this association in the present study. In addition, men who survived long enough to participate in the present study may have had a low likelihood of CIMT progression, and healthier participants within the cohort may have been more likely to have completed follow-up than men with increasing CIMT. The annual rate of CIMT progression in our study population (approximately 0.01 mm, or 10 µm/year) was similar to rates reported in MESA and other cohort studies including the ARIC study (Ranjit et al. 2006) and in older men and women in the Whitehall II study (Halcox et al. 2009).

We observed significant associations with the mean of ln-transformed daily predictions of black carbon at participant residence. In our secondary, exploratory analyses, we examined associations with measures of residential proximity to major roadways as well as traffic density. In the present study, contrary to our expectation, mean CIMT was greatest in men living farthest from a major road at baseline, although associations were not statistically significant and only a small number of participants (*n* = 32) lived ≤ 200 m from of a major road at baseline. A study by Allen et al. (2009) reported no association between roadway proximity and abdominal aortic artery calcification, another measure of atherosclerotic burden. As these authors have pointed out, the roadway classification system describes a type of road and not the traffic volume and traffic pollution, more directly, which may in part explain these differing results. In the present study, CIMT was higher in participants exposed to traffic counts higher than the first quartile, but estimates were relatively imprecise and not statistically significant.

Of the potential susceptibility factors that we examined, the only statistically significant interaction was observed for stratification by obesity: We observed higher associations with black carbon among non-obese individuals. We hypothesized that this finding was due to the strong association between BMI and CIMT in this study, such that obesity and other related factors likely have a larger effect on a measure of subclinical atherosclerosis than black carbon and the association between black carbon and intima-media thickness is observed only in the non-obese individuals. In addition, Freedman et al. (2004) have noted that CIMT may be more difficult to measure in obese individuals and may be assessed with less precision. The association between black carbon and CIMT was also somewhat stronger in men without diabetes than in men with diabetes, although estimates were imprecise. These results contrast those of other studies that have reported evidence to suggest that persons with diabetes are particularly susceptible to the effects of traffic pollution (Baja et al. 2010; Dubowsky et al. 2006; O’Neill 2005). Finally, previous studies have reported larger associations among statin users, which we did not detect although there may be confounding by indication and findings may reflect existing atherosclerotic risk factors that could also potentially increase susceptibility to air pollution. The results that we observed may differ from those observed in previous studies because of differences in exposure characterization, age range, and other population characteristics. We also cannot rule out the possibility that our findings of a significant association for BMI may be due to chance.

Our estimates of long-term black carbon exposure were model based, rather than direct measures from monitors. Use of exposure estimates based on residential address may misclassify personal exposure levels. However, because we are examining relatively recent long-term exposures and only 16% of the population changed residence over the course of follow-up, the lack of substantial occupational or commuting exposure to traffic-related air pollution in this largely retired cohort (93% completely retired or semiretired at baseline) suggests that residence-based exposure estimates may serve as a good proxy for personal exposure. In addition, our exposure is modeled on the log scale, which limits the influence of very high estimates of black carbon levels. On the other hand, this approach does not address the influence of very low exposure values. Whereas our model diagnostics suggested that the association was linear on the log scale, results from analyses examining the association on the natural scale suggested a nonlinear dose response and a negative slope at high levels, although these results were imprecise and the CIs were wide. Our black carbon model predicts lower levels of exposure outside the region within I-495, in which it was originally developed. Although we had considerably less power to detect an association in analyses restricted to within I-495 only (77% of participants), in these analyses we estimated stronger associations between black carbon levels and CIMT, but results were no longer statistically significant. It is possible, however, that the estimates we report in the full population underestimate the true association because of exposure misclassification, although we had considerably less power to detect an association in analyses restricted to within I-495 only. In addition, our analysis of annual black carbon concentrations may not fully account for long-term time trends because they were based on associations with annual average black carbon concentrations estimated for the year prior to baseline, which occurred between 2004 and 2008 and, therefore, may not fully account for long-term time trends. To attempt to address this, we also analyzed the associations assigning everyone black carbon exposure based on 2003 data for their addresses and the results were similar to those observed in our final model. Future work will be necessary to elucidate this association.

Because our study participants were elderly male residents of the Boston area, 97% of whom reported their race as white, these results may not be generalizable to other populations of environmentally exposed men and women. This particular analysis was also limited to a subset of Normative Aging Study participants who continued to be followed during the study period, many of whom consented to return on an additional day for CIMT testing. Our study participants were elderly men ranging in age from 61 to 96 years of age at first visit, who, on average, had already developed a considerable degree of atherosclerotic burden at baseline. Furthermore, the mean CIMT reported at baseline was higher than has been reported in other community-based samples, which generally have younger participants (Adar et al. 2013; Rivera et al. 2013). There may be some degree of measurement error in CIMT, but this would likely be nondifferential with respect to exposure. In addition, we also cannot rule out the role of residual confounding by socioeconomic position or other factors. Although there was some attrition over time, two or more measurements were obtained for 89% of individuals.

## Conclusions

We observed that low levels of ambient exposure to estimated black carbon were associated with CIMT in a population of elderly men continuing to participate in a long-term prospective cohort study. Given the growing interest in the relationship between air pollution and atherosclerosis (Künzli et al. 2011) and the limited body of human studies examining repeated measurement of indicators of atherosclerotic burden, future studies are needed to substantiate the association between specific sources of pollution and atherosclerotic progression in order to clarify the underlying mechanisms.

## Supplemental Material

(221 KB) PDF
