# Supplementary material for: Long-term Exposure to Black Carbon and Carotid Intima-Media Thickness: The Normative Aging Study
Source: Environ Health Perspect. 2013 Jul 2;121(9):1061–7. doi: 10.1289/ehp.1104845 (PMC3764069; doi:10.1289/ehp.1104845)
Supplement: (221 KB) PDF [file ehp.1104845.s001.pdf]

Environmental Health Perspectives doi:10.1289/ehp.1104845

Supplemental Material

Long-term Exposure to Black Carbon and Carotid Intima-Media Thickness: The Normative Aging Study

Elissa H. Wilker, Murray A. Mittleman, Brent A. Coull, Alexandros Gryparis, Michiel L. Bots, Joel Schwartz, and David Sparrow

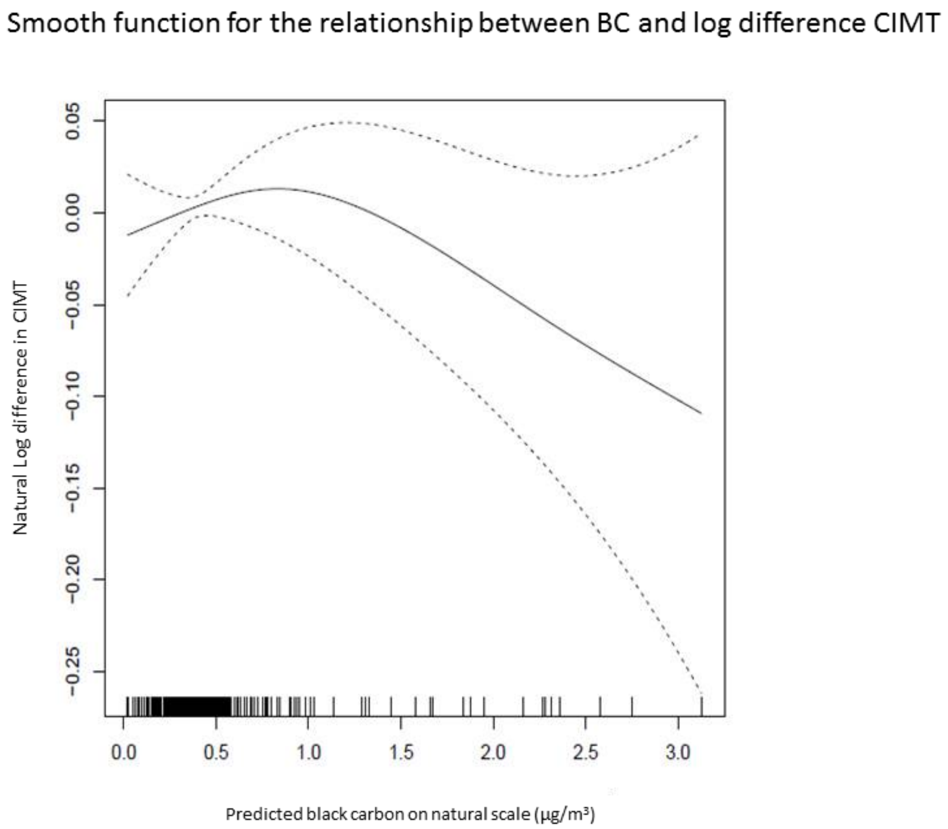

Supplemental Material, Figure S1. The association between BC and the natural log of CIMT was positive and linear at low levels, but has a negative slope with wide confidence intervals at higher levels where data are sparse.
